# MiR-21 binding site SNP within *ITGAM* associated with psoriasis susceptibility in women

**DOI:** 10.1371/journal.pone.0218323

**Published:** 2019-06-18

**Authors:** Pavel Hruska, Daniela Kuruczova, Vladimir Vasku, Julie Bienertova-Vasku

**Affiliations:** 1 Department of Pathological Physiology, Faculty of Medicine, Masaryk University, Brno, Czech Republic; 2 First Department of Dermatovenereology, St. Anne’s University Hospital Brno, Brno, Czech Republic; 3 Research Centre for Toxic Compounds in the Environment, Faculty of Science, Masaryk University, Brno, Czech Republic; Institut d'Investigacions Biomediques de Barcelona, SPAIN

## Abstract

**Background:**

Great progress has been made in the understanding of inflammatory processes in psoriasis. However, clarifying the role of genetic variability in processes regulating inflammation, including post-transcriptional regulation by microRNA (miRNA), remains a challenge.

**Objectives:**

We therefore investigated single nucleotide polymorphisms (SNPs) with a predicted change in the miRNA/mRNA interaction of genes involved in the psoriasis inflammatory processes.

**Methods:**

Studied SNPs rs2910164 C/G–*miR-146a*, rs4597342 T/C–*ITGAM*, rs1368439 G/T–*IL12B*, rs1468488 C/T–*IL17RA* were selected using a bioinformatics analysis of psoriasis inflammation-associated genes. These SNPs were then genotyped using a large cohort of women with psoriasis (n = 241) and healthy controls (n = 516).

**Results:**

No significant association with psoriasis was observed for rs2910164, rs1368439, and rs1468488 genotypes. However, the major allele T of rs4597342 –*ITGAM* was associated with approximately 28% higher risk for psoriasis in comparison to the patients with the C allele (OR = 1.28, 95% CI 1.01–1.61, p = 0.037). In case of genotypes, the effect of the T allele indicates the dominant model of disease penetrance as the CT and TT genotypes increase the chance of psoriasis up to 42% in comparison to CC homozygotes of rs4597342 (OR = 1.42, 95% CI = 1.05–1.94, p = 0.025).

**Conclusion:**

SNP rs4597342 in 3'UTR of *ITGAM* influencing miR-21 binding may be considered a risk factor for psoriasis development. Upregulated miR-21 in psoriasis is likely to inhibit CD11b production in the case of the rs4597342 T allele which may lead to Mac-1 dysfunction, resulting in an aberrant function of innate immune cells and leading to the production of cytokines involved in psoriasis pathogenesis.

## Introduction

Great progress has been made in the understanding of inflammatory processes involved in psoriasis pathogenesis in recent years. The interaction of genetic background and environmental factors is engaged in psoriasis development and influences the course of the disease. Characteristic inflammatory processes involve dendritic cells (DCs), Th1, Th17, Th22 lymphocytes, and other inflammatory cells. Cytokines produced by these cells support inflammation in the skin and induce abnormal behavior of keratinocytes which also contribute to the cytokine milieu and inflammatory circuits in psoriasis [1].

Inflammatory processes in psoriasis are influenced by a range of genetic, epigenetic and environmental factors. One such epigenetic factor are microRNAs (miRNA), small ~22 nucleotides long molecules of non-coding RNA, regulating gene expression at the post-transcriptional level by sequence-specific binding to the 3'UTR of target genes [2]. This fine-tuned tool regulates multiple target genes and significantly impacts a wide range of cellular processes including the development and behavior of inflammatory cell subsets, thereby affecting inflammatory response [3]. Since the discovery of deregulated miRNA expression in psoriasis, our knowledge of its contribution to psoriasis pathogenesis has widely expanded. The deregulated expression of miRNAs in psoriasis has been observed to be involved in the regulation of keratinocyte proliferation and differentiation while also influencing the regulation of inflammation in psoriatic skin [4–7]. Even though miRNA sequences are relatively evolutionary conserved, they can also exhibit a certain degree of genetic polymorphism. Recent studies have proven that single nucleotide polymorphisms (SNPs) in miRNA sequences may either alter sequence stability during maturation processes or change the sequence affinity to their target sites [8,9]. An example of such an SNP is rs2910164 within the miR-146a precursor molecule, which is localized directly in the miRNA seed site of miR-146a-3p [8,10]. Furthermore, even SNPs within the 3'UTR of a target gene itself can disrupt this otherwise perfectly tuned genetic regulation mechanism. This genetic variation in either the miRNA sequence or 3'UTR has been proven to be a risk factor for various diseases such as cancer [11,12], cardiovascular diseases [13,14], neuropathies [15] and chronic inflammatory diseases [16,17] including psoriasis [18]. Pivarcsi et al. proposed that genetic variation plays a key role in disrupting the balance of immune system regulation which consequently leads to chronic inflammation in psoriatic skin [19].

Considering the above-mentioned facts, we used a bioinformatics approach to identify SNPs within psoriasis inflammation-associated genes or miRNAs. As a result of this approach we selected and investigated the following genetic variations: SNP rs2910164 in miR-146a and SNPs rs4597342 in 3'UTR of *ITGAM* as an associated target of miR-21, rs1368439 in 3'UTR of *IL12B* as an associated target of miR-513a-5p and rs1468488 in 3'UTR of *IL17RA* as an associated target of miR-320a in psoriasis. These genetic variations–which may affect miRNAs’ post-transcriptional regulation of gene expression either through SNPs directly in the miRNA sequence, as in the case of SNP rs2910164 in miR-146a, or SNPs in 3'UTR of target genes–were assessed with respect to their potential association with psoriasis susceptibility.

## Materials and methods

### Patients and controls

This case-control study included only women with psoriasis (n = 241) and healthy women as controls (n = 516). Both cases and controls were obtained from the same source population of Central European origin with permanent residence in the South Moravian region of the Czech Republic. Men were excluded from the study due to unequal participation of men in the control group which was originally focused on dietary habits and lifestyle choices and involved volunteers–hence it suffered from strong selection bias towards the participation of women. All psoriasis patients (241 women; mean age 49.7 ± 16.4; mean age at psoriasis onset 25.9 ± 16.6) were diagnosed in accordance to criteria set up by American Academy of Dermatology and treated accordingly at the First Department of Dermatovenereology at St. Anne’s University Hospital Brno, Czech Republic. Three clinical subtypes of psoriasis were identified among the patients, i.e. plaque psoriasis (n = 183), pustular psoriasis (n = 12) and guttate psoriasis (n = 46). The control group consisted of healthy non-related individuals without a previous personal history of psoriasis or other severe skin disease (516 women; mean age 48.4 ± 13.93 years). This group was recruited at the Department of Preventive Medicine, Masaryk University, Brno. This study was approved by the Committee for Ethics of Medical Experiments on Human Subjects, Faculty of Medicine, Masaryk University, Brno, Czech Republic and performed in accordance with the Helsinki Declaration guidelines. All participants signed informed consent forms which were subsequently archived.

### Bioinformatics

Studied SNPs were selected using two different approaches. First, we considered miRNAs with altered expression in psoriasis; second, we searched for genes involved in the inflammatory processes of psoriasis. The list of candidate miRNAs involved in psoriasis pathogenesis was compiled following a search of relevant literature found on PubMed and Web of Science whereas the list of candidate genes associated with psoriasis pathogenesis was obtained using GWAS Integrator [20], Gene Prospector (unavailable since May 2016) [21] and DisGeNET [22]. All candidate miRNAs and genes were subject to bioinformatics analysis using the miRNASNP v2.0 online tool [23] which was designed to locate SNPs within miRNA sequences or the 3'UTR of target genes with predicted miRNA binding site gain or loss. Localized SNPs were ranked using Gibbs-free energy (ΔΔG) changes by comparing common alleles with their corresponding rare alleles. SNPs with the greatest change in binding energy were selected for experimental testing. The bioinformatics analysis led to the selection of four SNPs with the most promising effect on miRNA sequence stability or binding affinity: rs2910164 –*miR-146a*; rs4597342 –*ITGAM*, an associated target of miR-21-5p; rs1368439 –*IL12B*, an associated target of miR-513a-5p; rs1468488 –*IL17RA*, an associated target of miR-320a. The effects, location and other properties of selected SNPs are shown in **S1 Table**.

Additionally, the web-based application LDlink [24] was used to assess linkage disequilibrium (LD) of SNP rs4597342 to genetic variations previously associated with functional changes in *ITGAM*. This tool uses data from the 1000 Genomes Project Phase 3 (Version 5). Modules LDproxy and LDpair were used with CEU as the reference population.

### SNP genotyping

A standard PCR-RFLP analysis was used to determine all studied SNPs using the genomic DNA of all participants, extracted from peripheral blood. SNP rs2910164 in miR-146a was genotyped using a method previously employed by Zhang et al. [18]. The three remaining SNPs, i.e. rs4597342 in *ITGAM*, rs1368439 in *IL12B* and rs1468488 in *IL17RA*, were genotyped using carefully designed methods described in **S1 File**. The accuracy of genotyping data for SNPs obtained by PCR-RFLP analysis was further validated by Sanger sequencing of PCR products using newly designed sequencing primers with randomized sample selection.

### Statistical analysis

Statistical analysis was carried out using statistical software R (version 3.3.3) [25]. We initially explored the descriptive characteristics of the study population and the basic relationships between individual variables. All categorical variables were expressed as percentages and continuous variables as mean ± standard deviation (SD) unless otherwise stated. For further analysis, values of p < 0.05 were considered statistically significant. Pearson’s χ^2^ test and Fisher’s exact test were used to assess the Hardy–Weinberg equilibrium for all examined SNPs. Both allelic and genotype association between individual SNPs and psoriasis were tested using Pearson’s χ^2^ test with a permutation test correction for multiple testing [26]. For SNPs with a significant relationship to psoriasis, a logistic regression model was constructed with psoriasis diagnosis as the dependent variable and a given SNP and recruitment age as independent variables.

## Results

A total number of 757 subjects were genotyped for four candidate SNPs: rs2910164 –miR-146a, rs4597342 –ITGAM, rs1368439 –IL12B, rs1468488 –IL17RA. Based on Pearson’s χ^2^ test and Fisher’s exact test, all genotypes of SNPs rs2910164 –*miR-146a*, rs4597342 –*ITGAM* and rs1368439 –*IL12B* were in agreement with the Hardy–Weinberg equilibrium in cases and controls. However, as the distribution of SNP rs1468488 –*IL17RA* genotypes was in the Hardy–Weinberg disequilibrium (p = 0.038) for the control group, SNP rs1468488 was excluded from further analyses. All genotype and allele frequencies were then compared between cases and controls (**Table 1**). The only difference between genotype frequencies was observed at the edge of significance (χ^2^ = 5.08, df = 2, p = 0.079) for SNP rs4597342 –*ITGAM*. However, when applying the dominant model of disease penetrance for SNP rs4597342 genotypes CT and TT, we observed a significant difference between cases and controls (p = 0.029). A significant difference (p = 0.014) was also observed in case of allelic comparison.

**Table 1:** Genotype and allele analysis of studied SNPs.

SNP		Genotypes / Alleles	Cases (n = 241)	Controls (n = 516)	Odds ratio^#^ (95% CI)	P-value
**miR-146a rs2910164**		CC	12	29	ref.	0.669*
		CG	85	166	1.24 (0.60–2.55)	
		GG	144	321	1.08 (0.54–2.19)	
		C	109	224	1.13 (0.81–1.37)	0.329*
		G	373	808		
	D	GG / CG+CC	144 / 97	321 / 195	1.11 (0.81–1.52)	0.570**
	R	CC / CG+GG	12 / 229	29 / 487	1.14 (0.57–2.27)	0.863**
**ITGAM rs4597342**		CC	105	270	ref.	*0*.079*
		CT	110	201	1.41 (1.02–2.07)	
		TT	26	45	1.49 (0.87–2.53)	
		C	320	741	1.29 (1.02–1.63)	0.014*
		T	162	291		
	D	CC / CT+TT	105 / 136	270 / 246	1.42 (1.05–1.93)	0.029**
	R	TT / CT+CC	26 / 215	45 / 471	1.27 (0.76–2.11)	0.363**
**IL12B rs1368439**		GG	4	14	ref.	0.218*
		GT	80	142	1.97 (0.63–6.19)	
		TT	157	360	1.53 (0.50–4.71)	
		G	88	170	1.13 (0.85–1.50)	0.182*
		T	394	826		
	D	TT / GT+GG	157 / 84	360 / 156	1.24 (0.89–1.71)	0.209**
	R	GG / GT+TT	4 / 237	14 / 502	1.65 (0.54–5.07)	0.452**
**IL17RA rs1468488** ***		CC	10	25	ref.	0.558*
		CT	93	217	1.07 (0.49–2.31)	
		TT	138	274	1.26 (0.59–2.70)	
		C	113	267	-	-
		T	369	765		
	D	TT/CC+TC	138/274	103/242	1.18 (0.87–1.61)	0.309**
	R	CC/TC+TT	Oct-25	231/491	1.18 (0.56–2.49)	0.853**

D–Dominant model of disease penetrance; R–Recessive model of disease penetrance

^#^OR for risk allele/genotype is presented (for 2x2 tables)

*P-values of Pearson’s χ^2^ test with permutation test correction

**Fisher’s exact test P-values

***rs1468488 was in Hardy–Weinberg disequilibrium for the controls; the allelic analysis could not be performed

Logistic regression models with psoriasis diagnosis as a dependent variable were subsequently created in order to evaluate the risk of psoriasis for the T allele and CT+TT genotypes of SNP rs4597342. This logistic regression model showed significantly higher odds of psoriasis for the rs4597342 T allele regardless of subject recruitment age (OR = 1.28, 95% CI 1.01–1.61, p = 0.037). Considering the dominant model of disease penetrance, a significantly increased risk of psoriasis was observed for rs4597342 CT and TT genotypes compared to the CC genotype (OR = 1.42, 95% CI = 1.05–1.94, p = 0.025) (**Table 2**).

**Table 2:** Logistic regression model analysis of ITGAM rs4597342.

	Alleles / Genotypes	Cases (n = 241)	Controls (n = 516)	Odds ratio (95% CI)	P-value
Recruitment age*				1.01 (1.00–1.02)	0.302
Alleles*	C	320	741	ref.	
	T	162	291	1.28 (1.01–1.61)	0.037
Recruitment age**				1.01 (1.00–1.02)	0.293
Dominant model**	CC	105	270	ref.	
	CT+TT	136	246	1.42 (1.04–1.94)	0.026

*Logistic regression model with psoriasis diagnosis as the dependent variable, rs4597342 alleles, and recruitment age as independent variables.

**Logistic regression model with psoriasis diagnosis as dependent variable, rs4597342 genotypes in dominant model, and recruitment age as independent variables.

Additionally, the role of each SNP in psoriasis subtypes was also tested. However, some categories did not meet the requirements for the lowest expected frequencies for statistical testing; this analysis requires a larger sample of psoriasis subtypes (**S2 Table**). We also conducted an analysis of genetic susceptibility in case subgroups based on the age of psoriasis onset. Early-onset psoriasis (≤40 years) and late-onset psoriasis (>40 years) are known to have different genetic backgrounds and clinical patterns in a Caucasian population [27]. Therefore, the cohort of cases was divided into two groups, i.e. early onset (≤40 years, n = 196) and late onset (>40 years, n = 45), and subsequently analyzed for genetic susceptibility to psoriasis in the context of the family history of psoriasis and each of the studied SNPs. Performed analyses suggest a significantly higher chance of psoriasis with early onset in case of rs2910164 –*miR-146a* G allele (OR = 1.73, 95% CI 1.04–2.88, p = 0.037), however, this result should be interpreted with caution and/or confirmed by independent experiment due to the possibility of type I error as we did not correct for multiple tests with this subanalysis (**S3 Table**).

Although we established a significant association of rs4597342 with psoriasis in women, it is important to note that the functional association with miR-21 binding is based only on *in silico* analysis. As a result, we decided to consider other functional genetic variants in LD to rs4597342. Therefore, we conducted a search of SNPs in LD to rs4597342 using the LDlink application. Following this, SNPs with R^2^ values ranging from 0.8 to 1.0 were searched for previous associations in published research. However, none of 46 SNPs within the range were previously described with a functional change in CD11b. In addition, we also evaluated SNPs known to change CD11b function and established their LD to rs4597342. Among the most discussed non-synonymous SNPs within ITGAM were rs1143678, rs1143679, and rs1143683. These SNPs were found in a strong LD and translate into missense mutations P1146S, R77H, and A589V, respectively, and affect Mac-1 functions [28]. A total LD was also observed between rs4597342 and rs1143678 (D' = 1, R^2^ = 0.085, p < 0.001), rs1143679 (D' = 1, R^2^ = 0.051, p = 0.001), and rs1143683 (D' = 1, R^2^ = 0.085, p < 0.001). Even though the allele frequencies are not the same, haplotype mapping suggests that the minor alleles of non-synonymous SNPs are not co-inherited with the rs4597342 T allele within the same haplotype.

## Discussion

In order to build on previous knowledge, we searched the miRNASNP v 2.0 database for SNPs that may cause alterations in the mechanism of post-transcriptional regulation by miRNA and thus contribute to psoriasis pathogenesis. Throughout this bioinformatics process, we strictly focused on genes and miRNAs involved in psoriasis inflammation. This analysis led us to select the following SNPs: rs2910164 C/G in miR-146a, rs4597342 T/C in the 3'UTR of *ITGAM*, rs1368439 G/T in the 3'UTR of *IL12B* and rs1468488 C/T in 3'UTR of *IL17RA*. The majority of other genes involved in psoriasis inflammation rarely exhibit polymorphisms in the 3'UTR or the change in the Gibbs-free energy due to the polymorphism is low.

Polymorphic miR-146a, one of the most abundantly expressed miRNAs in psoriatic skin, has been suggested to be involved in the regulation of innate immunity and proliferation of keratinocytes [6,7,29,30]. The functional SNP rs2910164 C/G in miR-146a was previously associated with an increased risk of psoriasis in the Chinese Han population. In the study, the rs2910164 G allele was shown to exhibit lower levels of mature miR-146a that impair its regulation on the expression of *EGFR*, resulting in the increased proliferation of keratinocytes [18]. Additionally, the most recent study by Srivastava et al. describes a lower sensitivity to IL-17-mediated skin inflammation in case of the rs2910164 CC genotype that indicates a protective character in psoriasis [31]. Although we did not observe any significant association of rs2910164 with psoriasis when comparing the cases to controls, we have found a significant association with early psoriasis onset (≤40 years). It could be suggested that early-onset psoriasis and late-onset psoriasis manifest themselves with distinct clinical features and may be caused by slightly different pathogenic pathways [27].

rs4597342 is localized it the 3'UTR of *ITGAM* coding CD11b, the α-chain of integrin receptor CD11b/CD18 also known as Mac-1 or CR3. This receptor is highly expressed primarily on the surface of innate immune cells such as monocytes, macrophages, neutrophils, and DCs. CD11b modulates cell adhesion, migration, tissue recruitment and phagocytosis of innate immune cells, and also modulates signaling pathways in these cells, e.g. the Toll-like receptor (TLR) signaling pathways [32,33]. While CD11b is present in an inactive conformation on circulating leukocytes under basal conditions, it is rapidly activated following TLR stimulation. Its activation reduces the activation of NFκB that consequently leads to the reduced production of IL-6 and other pro-inflammatory cytokines. On the other hand, innate cells deficient in CD11b result in the enhanced activation of NFκB and other TLR-dependent pathways and inflammatory cytokine production [32,34]. CD11b was also associated with the negative regulation of other immune cell signaling pathways following ligand binding, e.g. suppression of T-cell activation [35], negative regulation of Th17 cell development [36,37], and modulation of DC maturation and function [34]. The impairment in CD11b may thus significantly influence the inflammatory processes in psoriasis.

**Fig 1:**
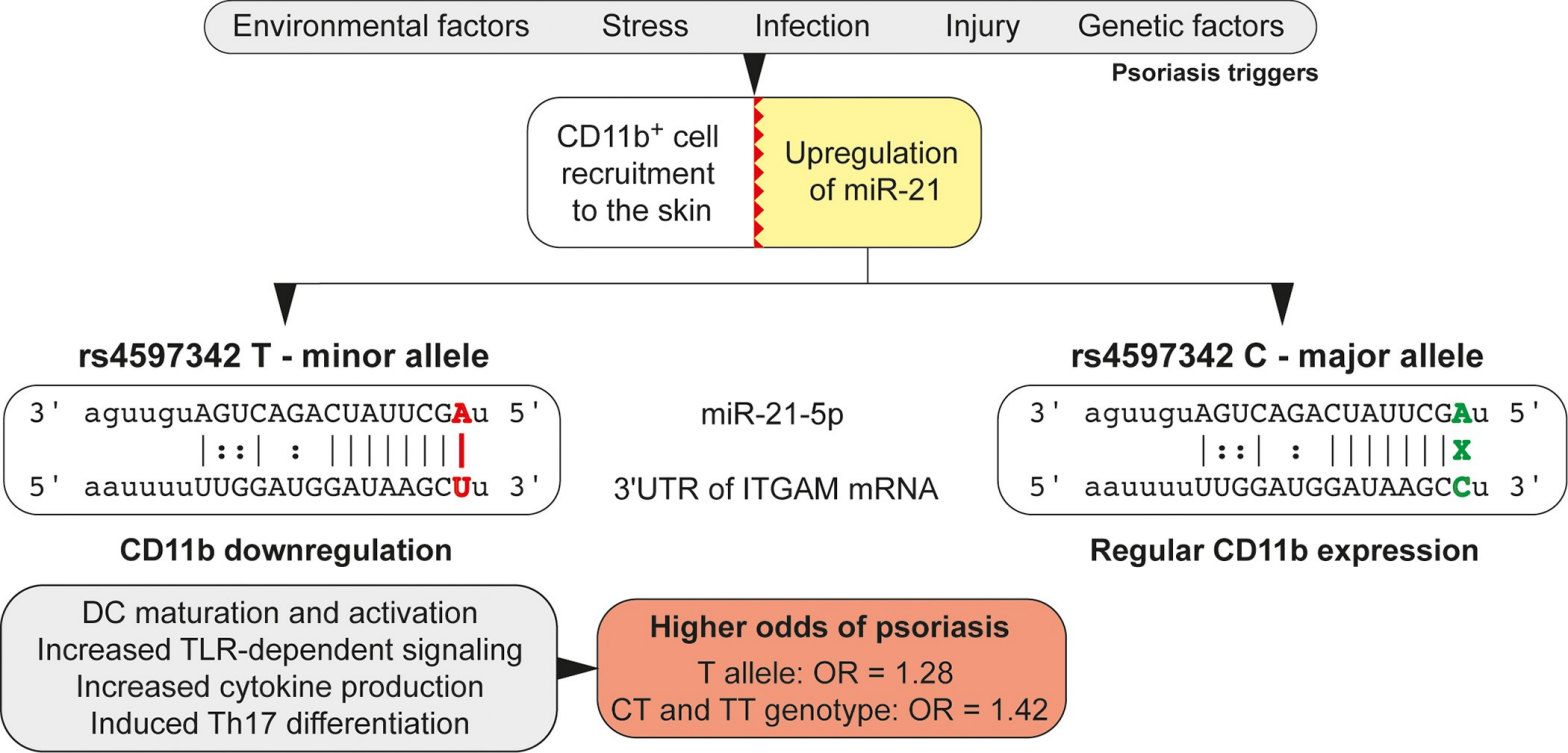
rs4597342 proposed mechanism of psoriasis susceptibility.

In this study we have investigated the SNP rs4597342 as a possible target of miR-21 that is upregulated in psoriasis [5,6]. Since the rs4597342 T allele is associated with a binding site gain for miR-21, the expression of *ITGAM* can be negatively regulated and its deficiency may result in the CD11b/CD18 receptor dysfunction. We have shown that the T allele and CT and TT genotypes are significantly associated with higher risk of psoriasis in women; therefore, our hypothesis which associates CD11b deficiency with miR-21 post-transcriptional regulation in psoriasis might be plausible (**Fig 1**). Some features of CD11b deficiency like enhanced activation of NFκB and other TLR-dependent pathways [38], Th-17 cells expansion [1], higher activity of DCs and production of some pro-inflammatory cytokines, e.g. IL-1β, IL-6, IL-12, IL-23, and TNF-α [39], are observed in psoriasis. Additionally, CD11^+^ cells are accumulated at the site of inflammation in psoriasis and the Mac-1 receptor is utilized for cell migration into the tissue [40]. Nevertheless, blocking the Mac-1 receptor does not result in complete inhibition of innate cell adhesion and migration [41]; as a result, CD11b deficiency may not affect cell migration while still affecting the remaining above-mentioned inflammatory properties of innate immune cells. However, the role of the Mac-1 receptor in psoriasis has not been properly studied and information about CD11b in psoriasis patients is very limited. Thus far, only a study by van Pelt *et al*. [42] observed a decreased expression of CD11b on circulating polymorphonuclear leukocytes in patients with plaque psoriasis in comparison to healthy controls. While the authors were unable to explain this observation, it might in fact be explained by our results. On the other hand, other studies comparing psoriasis patients with healthy controls did not report any significant differences for CD11b levels [41,43]. However, Sjögren *et al*. [43] reported higher levels of CD11b in patients with pustular psoriasis in comparison with those with plaque psoriasis, and suggested a different mechanism for pustular psoriasis development involving CD11b expression.

Moreover, several non-synonymous SNPs localized within *ITGAM* have been previously associated with systemic lupus erythematosus (SLE) susceptibility [28,33,44]. Although our LD analysis suggests that the rare variants of the three most common *ITGAM* SNPs, i.e. rs1143678, rs1143679 and rs1143683, are not within the same haplotype as the rs4597342 T allele, SLE and psoriasis do share some inflammatory features which might be associated with functionally deficient CD11b as a result of these SNPs. All three variants across different protein domains result in a functional deficiency of CD11b with limited effects on its surface expression levels [28,33]. However, as these SNPs have not been studied in psoriasis, we cannot discuss the involvement of these variants in psoriasis pathogenesis.

Genetic variability in the area of *IL12B* and *IL17RA* has previously been associated with psoriasis in many studies. The products of these genes have been found to contribute to psoriasis pathogenesis and to influence treatment response [45–48]. Therefore, we investigated SNP rs1368439 G/T in 3'UTR of *IL12B* where its minor allele G was associated with the gain of a binding site for miR-513a-5p. This interaction could potentially lower the *IL12B* expression and thus alleviate inflammatory processes during psoriasis. Nevertheless, we did not observe any significant association of rs1368439 with psoriasis. Similarly, no association was found in the case of SNP rs1468488 C/T in 3'UTR of *IL17RA* with the loss of a binding site for miR-320a, which could result in the higher expression of IL-17RA and thus influence the IL-17 pathways in psoriasis. However, no robust conclusion can be derived on this SNP as the control group was in a Hardy–Weinberg disequilibrium. The results of the initial genotyping method were confirmed by DNA sequencing, hence the possible explanation may be the selection bias that could had been introduced by the control group study design which explored dietary habits and lifestyle choices in general population and hence may be inadvertently biased towards specific phenotypes.

Although the studied SNPs were chosen following a rigorous bioinformatics analysis, it is important to note that all miRNA/SNP interactions were only predicted *in silico* and should thus be validated experimentally. Also, as the analysis only focused on women, it is important to confirm the results in man, especially in the case of *ITGAM* rs4597342. The variability of *ITGAM* has been previously associated with SLE which has a nine times higher prevalence in women [33]; it is thus possible that the effect of rs4597342 in psoriasis may be sex-dependent. Moreover, while it is likely that studied SNPs do not pose a causal risk for psoriasis development, they may still alter the severity and the course of psoriasis development–which we were unable to uncover since no patient follow-up was included in this study and only DNA samples were available for analysis.

In conclusion, this study associated SNP rs4597342 within 3'UTR of *ITGAM* with psoriasis susceptibility. The associated risk was observed for rs4597342 allele T which is linked to the gain of a binding site for miR-21, known to be upregulated during psoriasis. The genotype analysis suggests a dominant model of disease penetrance with approximately 42% higher odds of psoriasis for rs4597342 CT and TT genotypes. The T allele together with overexpression of miR-21 may cause CD11b deficiency on innate immune cells. This CD11b deficiency may not limit cell migration but can disrupt signaling pathways such as TLR-dependent pathways, stimulates Th-17 expansion, and increases DCs activity, all of which contribute to psoriasis pathogenesis. However, this functional hypothesis must be experimentally tested *in vitro* and/or *in vivo*.

## Supporting information

S1 Table SNP characteristics. (DOCX)

S2 Table SNP analysis of psoriasis subtypes. (DOCX)

S3 Table Early and late onset psoriasis SNP association analysis. (DOCX)

S1 File SNP genotyping methods. (PDF)

S1 Dataset Raw data. (XLSX)

## References

[1] Lowes MA, Russell CB, Martin DA, Towne JE, Krueger JG. The IL-23/T17 pathogenic axis in psoriasis is amplified by keratinocyte responses. Trends in Immunology. 2013;34: 174–181. 10.1016/j.it.2012.11.005 23291100 PMC3721313

[2] Bartel DP. MicroRNAs: Genomics, Biogenesis, Mechanism, and Function. Cell. 2004;116: 281–297. 14744438 10.1016/s0092-8674(04)00045-5

[3] Lindsay MA. microRNAs and the immune response. Trends in Immunology. 2008;29: 343–351. 10.1016/j.it.2008.04.004 18515182

[4] Guinea-Viniegra J, Jiménez M, Schonthaler HB, Navarro R, Delgado Y, Concha-Garzón MJ, et al Targeting miR-21 to treat psoriasis. Sci Transl Med. 2014;6: 225re1 10.1126/scitranslmed.3008089 24574341

[5] Meisgen F, Xu N, Wei T, Janson PC, Obad S, Broom O, et al MiR-21 is up-regulated in psoriasis and suppresses T cell apoptosis: Letter to the Editor. Experimental Dermatology. 2012;21: 312–314. 10.1111/j.1600-0625.2012.01462.x 22417311

[6] Sonkoly E, Wei T, Janson PCJ, Sääf A, Lundeberg L, Tengvall-Linder M, et al MicroRNAs: Novel Regulators Involved in the Pathogenesis of Psoriasis? Zimmer J, editor. PLoS ONE. 2007;2: e610 10.1371/journal.pone.0000610 17622355 PMC1905940

[7] Zibert JR, Løvendorf MB, Litman T, Olsen J, Kaczkowski B, Skov L. MicroRNAs and potential target interactions in psoriasis. Journal of Dermatological Science. 2010;58: 177–185. 10.1016/j.jdermsci.2010.03.004 20417062

[8] Jazdzewski K, Murray EL, Franssila K, Jarzab B, Schoenberg DR, Chapelle A de la. Common SNP in pre-miR-146a decreases mature miR expression and predisposes to papillary thyroid carcinoma. PNAS. 2008;105: 7269–7274. 10.1073/pnas.0802682105 18474871 PMC2438239

[9] Sethupathy P, Collins FS. MicroRNA target site polymorphisms and human disease. Trends in Genetics. 2008;24: 489–497. 10.1016/j.tig.2008.07.004 18778868

[10] Xu B, Feng N-H, Li P-C, Tao J, Wu D, Zhang Z-D, et al A functional polymorphism in Pre-miR-146a gene is associated with prostate cancer risk and mature miR-146a expression in vivo. Prostate. 2010;70: 467–472. 10.1002/pros.21080 19902466

[11] Brendle A, Lei H, Brandt A, Johansson R, Enquist K, Henriksson R, et al Polymorphisms in predicted microRNA-binding sites in integrin genes and breast cancer: ITGB4 as prognostic marker. Carcinogenesis. 2008;29: 1394–1399. 10.1093/carcin/bgn126 18550570

[12] Landi D, Gemignani F, Naccarati A, Pardini B, Vodicka P, Vodickova L, et al Polymorphisms within micro-RNA-binding sites and risk of sporadic colorectal cancer. Carcinogenesis. 2008;29: 579–584. 10.1093/carcin/bgm304 18192692

[13] Buraczynska M, Zukowski P, Wacinski P, Ksiazek K, Zaluska W. Polymorphism in microRNA-196a2 contributes to the risk of cardiovascular disease in type 2 diabetes patients. J Diabetes Complications. 2014;28: 617–620. 10.1016/j.jdiacomp.2014.05.006 24972764

[14] Liu M-E, Liao Y-C, Lin R-T, Wang Y-S, Hsi E, Lin H-F, et al A functional polymorphism of PON1 interferes with microRNA binding to increase the risk of ischemic stroke and carotid atherosclerosis. Atherosclerosis. 2013;228: 161–167. 10.1016/j.atherosclerosis.2013.01.036 23497787

[15] Ciccacci C, Morganti R, Di Fusco D, D’Amato C, Cacciotti L, Greco C, et al Common polymorphisms in MIR146a, MIR128a and MIR27a genes contribute to neuropathy susceptibility in type 2 diabetes. Acta Diabetol. 2014;51: 663–671. 10.1007/s00592-014-0582-2 24682535

[16] Brest P, Lapaquette P, Souidi M, Lebrigand K, Cesaro A, Vouret-Craviari V, et al A synonymous variant in IRGM alters a binding site for miR-196 and causes deregulation of IRGM-dependent xenophagy in Crohn’s disease. Nature Genet. 2011;43: 242–U24. 10.1038/ng.762 21278745

[17] Lofgren SE, Frostegard J, Truedsson L, Pons-Estel BA, D’Alfonso S, Witte T, et al Genetic association of miRNA-146a with systemic lupus erythematosus in Europeans through decreased expression of the gene. Genes Immun. 2012;13: 268–274. 10.1038/gene.2011.84 22218224 PMC3640319

[18] Zhang W, Yi X, Guo S, Shi Q, Wei C, Li X, et al A single-nucleotide polymorphism of miR-146a and psoriasis: an association and functional study. J Cell Mol Med. 2014;18: 2225–2234. 10.1111/jcmm.12359 25209759 PMC4224556

[19] Pivarcsi A, Ståhle M, Sonkoly E. Genetic polymorphisms altering microRNA activity in psoriasis–a key to solve the puzzle of missing heritability? Exp Dermatol. 2014;23: 620–624. 10.1111/exd.12469 24917490

[20] Yu W, Yesupriya A, Wulf A, Hindorff LA, Dowling N, Khoury MJ, et al GWAS Integrator: a bioinformatics tool to explore human genetic associations reported in published genome-wide association studies. Eur J Hum Genet. 2011;19: 1095–1099. 10.1038/ejhg.2011.91 21610748 PMC3190251

[21] Yu W, Wulf A, Liu T, Khoury MJ, Gwinn M. Gene Prospector: An evidence gateway for evaluating potential susceptibility genes and interacting risk factors for human diseases. BMC Bioinformatics. 2008;9: 528 10.1186/1471-2105-9-528 19063745 PMC2613935

[22] Piñero J, Queralt-Rosinach N, Bravo À, Deu-Pons J, Bauer-Mehren A, Baron M, et al DisGeNET: a discovery platform for the dynamical exploration of human diseases and their genes. Database (Oxford). 2015;2015. 10.1093/database/bav028 PMC4397996 25877637

[23] Gong J, Liu C, Liu W, Wu Y, Ma Z, Chen H, et al An update of miRNASNP database for better SNP selection by GWAS data, miRNA expression and online tools. Database (Oxford). 2015;2015. 10.1093/database/bav029 PMC4397995 25877638

[24] Machiela MJ, Chanock SJ. LDlink: a web-based application for exploring population-specific haplotype structure and linking correlated alleles of possible functional variants. Bioinformatics. 2015;31: 3555–3557. 10.1093/bioinformatics/btv402 26139635 PMC4626747

[25] R Core Team (2013). R: A language and environment for statistical computing. R Foundation for Statistical Computing, Vienna, Austria URL http://www.R-project.org/.

[26] Zhang L, Jiang Y. MCPerm: A Monte Carlo permutation method for multiple test correlation. R package version 1.1. 4. 2013.

[27] Theodorakopoulou E, Yiu ZZN, Bundy C, Chularojanamontri L, Gittins M, Jamieson LA, et al Early- and late-onset psoriasis: a cross-sectional clinical and immunocytochemical investigation. Br J Dermatol. 2016;175: 1038–1044. 10.1111/bjd.14886 27459949

[28] Zhou Y, Wu J, Kucik DF, White NB, Redden DT, Szalai AJ, et al Multiple Lupus Associated ITGAM Variants Alter Mac-1 Function on Neutrophils. Arthritis Rheum. 2013;65: 2907–2916. 10.1002/art.38117 23918739 PMC3969028

[29] Meisgen F, Xu Landén N, Wang A, Réthi B, Bouez C, Zuccolo M, et al MiR-146a Negatively Regulates TLR2-Induced Inflammatory Responses in Keratinocytes. The Journal of Investigative Dermatology. 2014;134: 1931–40. 10.1038/jid.2014.89 24670381

[30] Raaby L, Langkilde A, Kjellerup R b., Vinter H, Khatib S h., Hjuler K f., et al Changes in mRNA expression precede changes in microRNA expression in lesional psoriatic skin during treatment with adalimumab. Br J Dermatol. 2015;173: 436–447. 10.1111/bjd.13721 25662483

[31] Srivastava A, Nikamo P, Lohcharoenkal W, Li D, Meisgen F, Xu Landén N, et al MicroRNA-146a suppresses IL-17-mediated skin inflammation and is genetically associated with psoriasis. J Allergy Clin Immunol. 2017;139: 550–561. 10.1016/j.jaci.2016.07.025 27568078

[32] Han C, Jin J, Xu S, Liu H, Li N, Cao X. Integrin CD11b negatively regulates TLR-triggered inflammatory responses by activating Syk and promoting degradation of MyD88 and TRIF via Cbl-b. Nature Immunology. 2010;11: 734–742. 10.1038/ni.1908 20639876

[33] Khan SQ, Khan I, Gupta V. CD11b Activity Modulates Pathogenesis of Lupus Nephritis. Front Med. 2018;5. 10.3389/fmed.2018.00052 PMC5862812 29600248

[34] Škoberne M, Somersan S, Almodovar W, Truong T, Petrova K, Henson PM, et al The apoptotic-cell receptor CR3, but not αvβ5, is a regulator of human dendritic-cell immunostimulatory function. Blood. 2006;108: 947–955. 10.1182/blood-2005-12-4812 16614246 PMC1895855

[35] Varga G, Balkow S, Wild MK, Stadtbaeumer A, Krummen M, Rothoeft T, et al Active MAC-1 (CD11b/CD18) on DCs inhibits full T-cell activation. Blood. 2007;109: 661–669. 10.1182/blood-2005-12-023044 17003381

[36] Ehirchiou D, Xiong Y, Xu G, Chen W, Shi Y, Zhang L. CD11b facilitates the development of peripheral tolerance by suppressing Th17 differentiation. J Exp Med. 2007;204: 1519–1524. 10.1084/jem.20062292 17562817 PMC2118631

[37] Nowatzky J, Manches O, Khan SA, Godefroy E, Bhardwaj N. Modulation of human Th17 cell responses through complement receptor 3 (CD11 b/CD18) ligation on monocyte-derived dendritic cells. Journal of Autoimmunity. 2018;92: 57–66. 10.1016/j.jaut.2018.05.005 29908907 PMC6498856

[38] Chen J-Q, Szodoray P, Zeher M. Toll-Like Receptor Pathways in Autoimmune Diseases. Clinic Rev Allerg Immunol. 2016;50: 1–17. 10.1007/s12016-015-8473-z 25687121

[39] Lowes MA, Suárez-Fariñas M, Krueger JG. Immunology of Psoriasis. Annual Review of Immunology. 2014;32: 227–255. 10.1146/annurev-immunol-032713-120225 24655295 PMC4229247

[40] van Pelt JPA, Kuijpers SHH, van de Kerkhof PCM, de Jong EMGJ. The CD11bCD18-Integrin in the pathogenesis of psoriasis. Journal of Dermatological Science. 1998;16: 135–143. 9459126 10.1016/s0923-1811(97)00041-8

[41] Wetzel A, Wetzig T, Haustein UF, Sticherling M, Anderegg U, Simon JC, et al Increased Neutrophil Adherence in Psoriasis: Role of the Human Endothelial Cell Receptor Thy-1 (CD90). Journal of Investigative Dermatology. 2006;126: 441–452. 10.1038/sj.jid.5700072 16374458

[42] Van Pelt JPA, De Jong EMGJ, Van Erp PEJ, Van De Kerkhof PCM. Decreased CD11b expression on circulating polymorphonuclear leukocytes in patients with extensive plaque psoriasis. EJD European journal of dermatology. 1997;7: 324–328.

[43] Sjögren F, Ljunghusen O, Baas A, Coble BI, Stendahl O. Expression and Function of ∼ 2 Integrin CD11BCD18 on Leukocytes from Patients with Psoriasis. ACTA DERMATOVENEREOLOGICA-STOCKHOLM. 1999;79: 105–110. 10.1080/000155599750011291 10228626

[44] Fan Y, Li L-H, Pan H-F, Tao J-H, Sun Z-Q, Ye D-Q. Association of ITGAM polymorphism with systemic lupus erythematosus: a meta-analysis. Journal of the European Academy of Dermatology and Venereology. 2011;25: 271–275. 10.1111/j.1468-3083.2010.03776.x 20629846

[45] Batalla A, Coto E, Gómez J, Eirís N, González-Fernández D, Gómez-De Castro C, et al IL17RA gene variants and anti-TNF response among psoriasis patients. Pharmacogenomics J. 2016; 10.1038/tpj.2016.70 27670766

[46] Cargill M, Schrodi SJ, Chang M, Garcia VE, Brandon R, Callis KP, et al A Large-Scale Genetic Association Study Confirms IL12B and Leads to the Identification of IL23R as Psoriasis-Risk Genes. Am J Hum Genet. 2007;80: 273–390. 10.1086/511051 17236132 PMC1785338

[47] Ellinghaus E, Ellinghaus D, Stuart PE, Nair RP, Debrus S, Raelson JV, et al Genome-wide association study identifies a psoriasis susceptibility locus at TRAF3IP2. Nat Genet. 2010;42: 991–995. 10.1038/ng.689 20953188 PMC3136364

[48] Nair RP, Ruether A, Stuart PE, Jenisch S, Tejasvi T, Hiremagalore R, et al Polymorphisms of the IL12B and IL23R genes are associated with psoriasis. The Journal Of Investigative Dermatology. 2008;128: 1653–1661. 10.1038/sj.jid.5701255 18219280 PMC2739284

