# Supplementary material for: MiR-21 binding site SNP within ITGAM associated with psoriasis susceptibility in women
Source: PLoS One. 2019 Jun 18;14(6):e0218323. doi: 10.1371/journal.pone.0218323 (PMC6581264; doi:10.1371/journal.pone.0218323)
Supplement: S1 Table — (DOCX) [file pone.0218323.s001.docx]

**S1 Table.** **SNP characteristics**

| **SNP ID** | **miRNA** | **Chr** | **Alleles** | **MAF (EUR)** | **Location** | **Target gain** | **Target loss** |
| --- | --- | --- | --- | --- | --- | --- | --- |
| rs2910164 | miR-146a-3p | 5 | C/G | C = 0.23 | In seed | 1,423 | 1,762 |
| **SNP ID** | **Gene** | **Chr** | **Alleles** | **MAF (EUR)** | **miRNA** | **SNP effect** | **ΔΔG (kcal/mol)** |
| rs4597342 | *ITGAM* | 16 | T/C | T = 0.33 | miR-21-5p | Gain | -18.30 |
| rs1368439 | *IL12B* | 5 | G/T | G = 0.17 | miR-513a-5p | Gain | -25.00 |
| rs1468488 | *IL17RA* | 22 | C/T | C = 0.29 | miR-320a | Loss | -30.50 |

Chr – chromosome; MAF – Minor allele frequency; Under “Target gain” and “Target loss” is a number of predicted targets with the gain or loss of binding site due to the SNP (the actual number is smaller, the database miRNASNP v2.0 is redundant).
