# Supplementary material for: MiR-21 binding site SNP within ITGAM associated with psoriasis susceptibility in women
Source: PLoS One. 2019 Jun 18;14(6):e0218323. doi: 10.1371/journal.pone.0218323 (PMC6581264; doi:10.1371/journal.pone.0218323)
Supplement: S2 Table — (DOCX) [file pone.0218323.s002.docx]

**S2 Table. SNP analysis of psoriasis subtypes**

|  | **Genotypes / alleles** | **Plaque psoriasis** | **Pustular psoriasis** | **Guttate psoriasis** | **P-value** |
| --- | --- | --- | --- | --- | --- |
| **miR-146a rs2910164** | CC | 8 | 2^#^ | 2 | - |
|  | GC | 66 | 3 | 16 |  |
|  | GG | 109 | 7 | 28 |  |
|  | C | 82 | 7 | 20 | 0.727 |
|  | G | 284 | 17 | 72 |  |
| **ITGAM rs4597342** | CC | 81 | 3 | 21 | 0.453 |
|  | CT | 84 | 8 | 18 |  |
|  | TT | 18 | 1 | 7 |  |
|  | C | 246 | 14 | 60 | 0.649 |
|  | T | 120 | 10 | 32 |  |
| **IL12B rs1368439** | GG | 4^#^ | 0^#^ | 0^#^ | - |
|  | TG | 61 | 5 | 14 |  |
|  | TT | 118 | 7 | 32 |  |
|  | G | 69 | 5 | 14 | 0.683 |
|  | T | 297 | 19 | 78 |  |
| **IL17RA rs1468488** | CC | 8 | 0^#^ | 2^#^ | - |
|  | CT | 66 | 7 | 20 |  |
|  | TT | 109 | 5 | 24 |  |
|  | C | 82 | 7 | 24 | 0.607 |
|  | T | 284 | 17 | 68 |  |

^#^Categories did not meet the requirements for the lowest expected frequencies for statistical testing.
