# Supplementary material for: MiR-21 binding site SNP within ITGAM associated with psoriasis susceptibility in women
Source: PLoS One. 2019 Jun 18;14(6):e0218323. doi: 10.1371/journal.pone.0218323 (PMC6581264; doi:10.1371/journal.pone.0218323)
Supplement: S3 Table — (DOCX) [file pone.0218323.s003.docx]

**S3 Table. Early and late onset psoriasis SNP association analysis**

|  |  |  | **Early* <=40** | **Late* >40** | **Odds ratio (95 % CI)** | **P-value** |
| --- | --- | --- | --- | --- | --- | --- |
|  | **Family history**** | yes | 68 | 11 | - | 0.220 |
|  |  | no | 128 | 34 |  |  |
| **miR-146a rs2910164** | **Genotypes** | CC | 8 | 4^#^ |  | - |
|  |  | CG | 65 | 20 | - |  |
|  |  | GG | 123 | 21 |  |  |
|  | **Allele** | C | 81 | 28 | 1.73 (1.04-2.88) | **0.037** |
|  |  | G | 311 | 62 |  |  |
|  | **Dominant model** | GG/ CG+CC | 123 / 73 | 21 / 24 | 1.93 (1.00-3.70) | 0.063 |
|  | **Recessive model** | CC / CG+GG | 8 / 188 | 4^#^ / 41 | 0.44 (0.13-1.52) | 0.245 |
| **ITGAM rs4597342** | **Genotypes** | CC | 86 | 19 |  | - |
|  |  | CT | 89 | 21 | - |  |
|  |  | TT | 21 | 5^#^ |  |  |
|  | **Allele** | C | 261 | 59 | 1.05 (0.65-1.70) | 0.870 |
|  |  | T | 131 | 31 |  |  |
|  | **Dominant model** | CC / CT+TT | 86 / 110 | 19 / 26 | 1.07 (0.56-2.06) | 0.870 |
|  | **Recessive model** | TT / CT+CC | 21 / 175 | 5 / 40 | 0.96 (0.34-2.70) | 1.000 |
| **IL12B rs1368439** | **Genotypes** | GG | 4^#^ | 0^#^ |  | - |
|  |  | GT | 62 | 18 | - |  |
|  |  | TT | 130 | 27 |  |  |
|  | **Allele** | G | 70 | 18 | 1.15 (0.65-2.05) | 0.650 |
|  |  | T | 322 | 72 |  |  |
|  | **Dominant model** | TT  GT+GG | 130 / 66 | 27 / 18 | 1.31 (0.67-2.56) | 0.488 |
|  | **Recessive model** | GG / GT+TT | 4^#^ / 192 | 0^#^ / 45 | - | - |
| **IL17RA rs1468488** | **Genotypes** | CC | 9 | 1^#^ |  | - |
|  |  | CT | 79 | 14 | - |  |
|  |  | TT | 108 | 30 |  |  |
|  | **Allele** | C | 74 | 16 | 0.65 (0.37-1.18) | 0.171 |
|  |  | T | 295 | 97 |  |  |
|  | **Dominant model** | TT / CT+CC | 108 / 88 | 30 / 15 | 0.61 (0.31-1.21) | 0.183 |
|  | **Recessive model** | CC / CT+TT | 9 / 187 | 1^#^ / 44 | - | - |

P-value was calculated by Fisher’s exact test

*Early onset was defined as patient age ≤ 40; late onset as patient age > 40.

**Patient’s parents or siblings were previously diagnosed with psoriasis.

^#^Categories did not meet the requirements for the lowest expected frequencies for statistical testing.
