# Supplementary material for: MiR-21 binding site SNP within ITGAM associated with psoriasis susceptibility in women
Source: PLoS One. 2019 Jun 18;14(6):e0218323. doi: 10.1371/journal.pone.0218323 (PMC6581264; doi:10.1371/journal.pone.0218323)
Supplement: S1 File — (PDF) [file pone.0218323.s004.pdf]

S1 File – SNP genotyping

A standard PCR-RFLP analysis was used to determine all studied SNPs using the genomic DNA of all participants, extracted from peripheral blood. SNP rs2910164 in miR-146a was genotyped using a method previously employed by Zhang et al. [18]. The three remaining SNPs, i.e. rs4597342 in *ITGAM*, rs1368439 in *IL12B* and rs1468488 in *IL17RA*, were genotyped using carefully designed methods described in **Table A**. The accuracy of genotyping data for SNPs obtained by PCR-RFLP analysis was validated by Sanger sequencing of PCR products using newly designed sequencing primers with randomized sample selection (**Table B**).

Table A - PCR-RFLP analysis

Gene	SNP ID	Primers 5'-3'	Product length (bp)	SNP position (bp)	RE**	RFLP fragments (bp)
miR-146a	rs2910164	F*: CATGGGTTGTGTCAGTGTCAGA**G**CT<br>R*: TGCCTTCTGTCTCCAGTCTTCCAA	147	23	*SacI*	CC: 25, 122<br>GG: 147
ITGAM	rs4597342	F: CAGCAGCTTCTCTCCACTGA<br>R: ACATCGCCTGGAATCTGTCT	368	234	*HindIII*	CC: 172, 196<br>TT: 58, 138, 172
IL12B	rs1368439	F: GCATCTGTCTGCTTCTC**C**CA<br>R: GCAATTTAGGGCCACTTACACT	506	21	*NcoI*	GG: 180, 326<br>TT: 20, 160, 326
IL17RA	rs1468488	F: GACACGATGGGGTCAGAGTC<br>R: GAGCCTCAAGGACGCTCTAC	293	73	*AluI*	CC: 26, 267<br>TT: 26, 71, 196

*F = forward primer, R = reverse primer; **Restriction endonucleases

Table B – PCR primers for sequencing

Gene	SNP ID	Primers 5'->3'	Product length (bp)
miR-146a	rs2910164	SeqF*: GGAGGGGTCTTTGCACCATC<br>SeqR: CCAGTCTTCCAAGCTCTTCAGC	198
ITGAM	rs4597342	SeqF: CGGTCAATCCTGTGGGTGAA<br>SeqR*: TTGCACATCGCCTGGAATCT	406
IL12B	rs1368439	SeqF*: CCGTAAGTGTCTGGAAGGCAAA<br>SeqR: TGTAATCACTTTACAGAGCGCACAT	254
IL17RA	rs1468488	F*: GACACGATGGGGTCAGAGTC<br>R: GAGCCTCAAGGACGCTCTAC	293

* used as sequencing primer (for IL17RA RFLP forward primer was used)

miR-146a rs2910164

a)

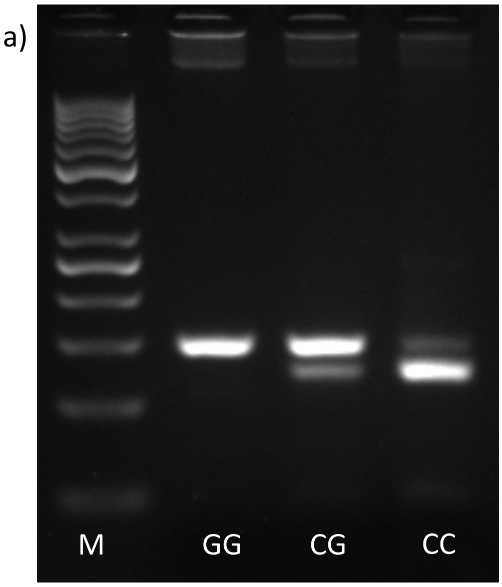

b)

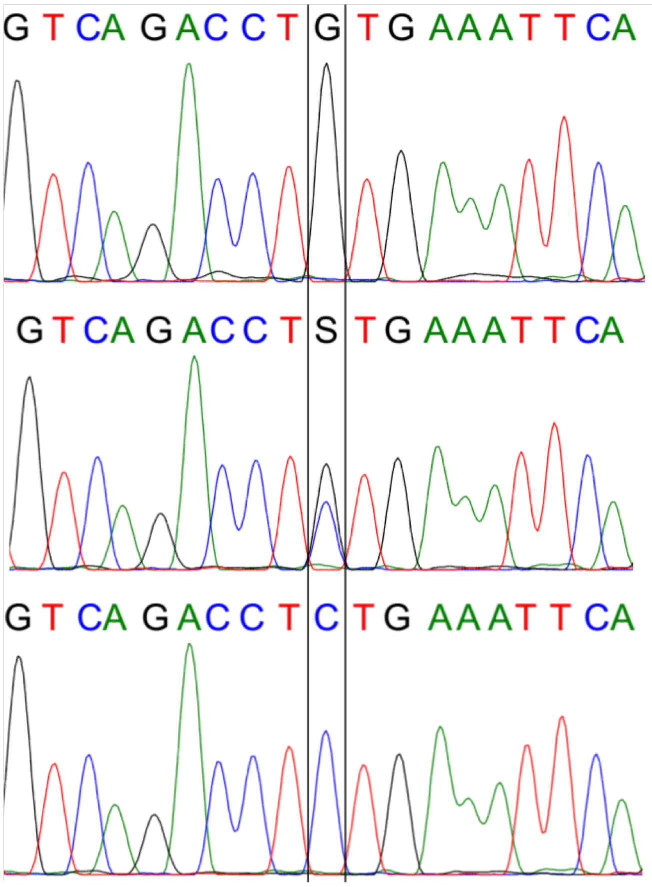

a) PCR-RFLP products on gel; M – GeneRuler 50 bp DNA Ladder (Thermo Scientific); genotypes

b) Genotypes obtained by Sanger sequencing

ITGAM rs4597342

a)

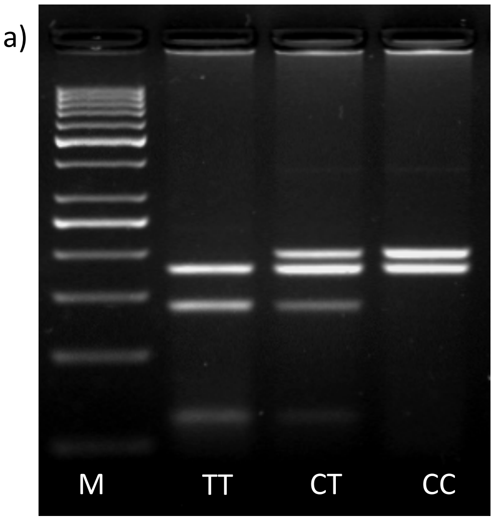

b)

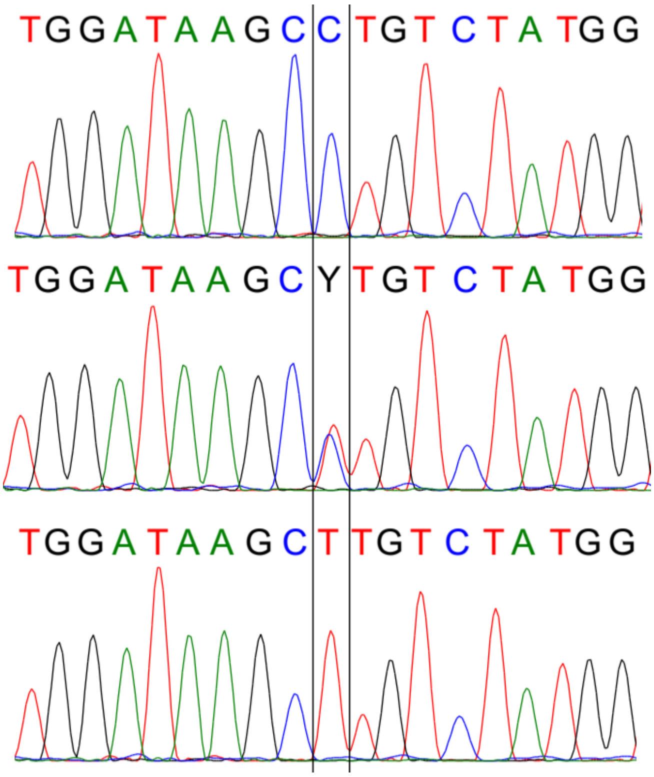

a) PCR-RFLP products on gel; M – GeneRuler 50 bp DNA Ladder (Thermo Scientific); genotypes

b) Genotypes obtained by Sanger sequencing - sequence shown in reverse complement

IL12B rs1368439

a)

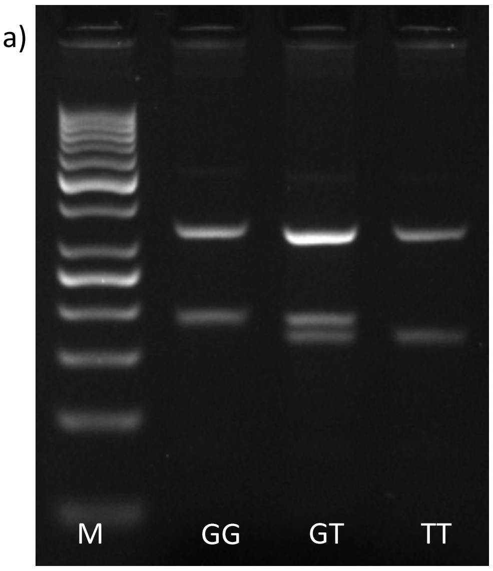

b)

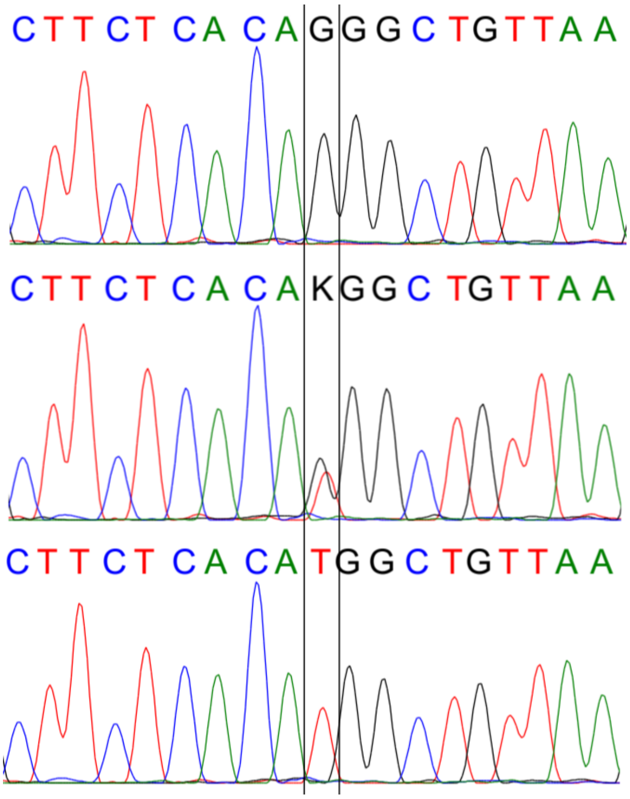

a) PCR-RFLP products on gel; M – GeneRuler 50 bp DNA Ladder (Thermo Scientific); genotypes

b) Genotypes obtained by Sanger sequencing - sequence shown in reverse complement

IL17RA rs1468488

a)

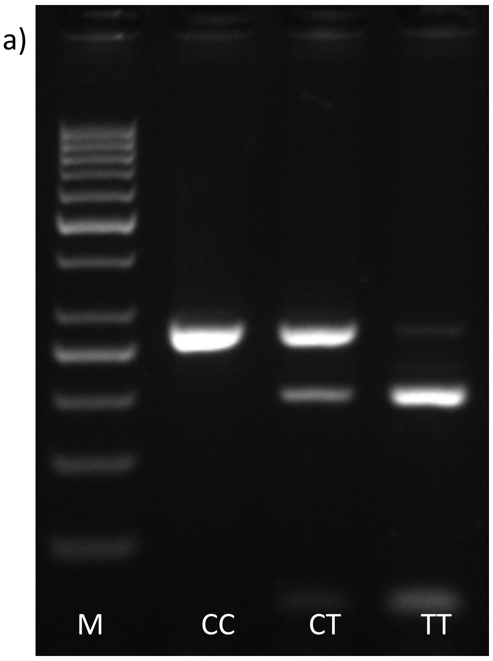

b)

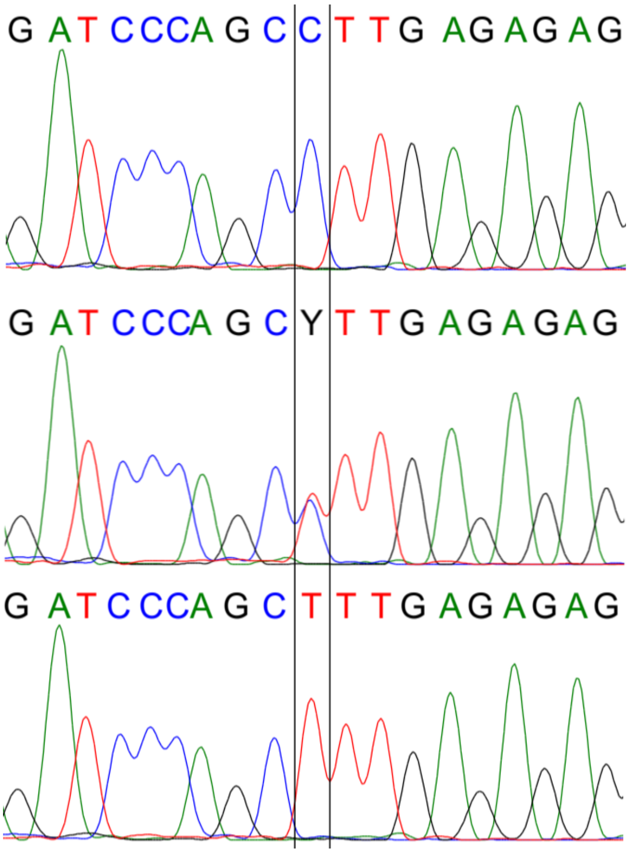

a) PCR-RFLP products on gel; M – GeneRuler 50 bp DNA Ladder (Thermo Scientific); genotypes

b) Genotypes obtained by Sanger sequencing
